# Supplementary material for: Chromatin organizer SATB1 controls the cell identity of CD4+ CD8+ double-positive thymocytes by regulating the activity of super-enhancers
Source: Nat Commun. 2022 Sep 22;13:5554. doi: 10.1038/s41467-022-33333-6 (PMC9500044; doi:10.1038/s41467-022-33333-6)
Supplement: Supplementary file 1 — Supplementary Information [file 41467_2022_33333_MOESM1_ESM.pdf]

Supplementary figures

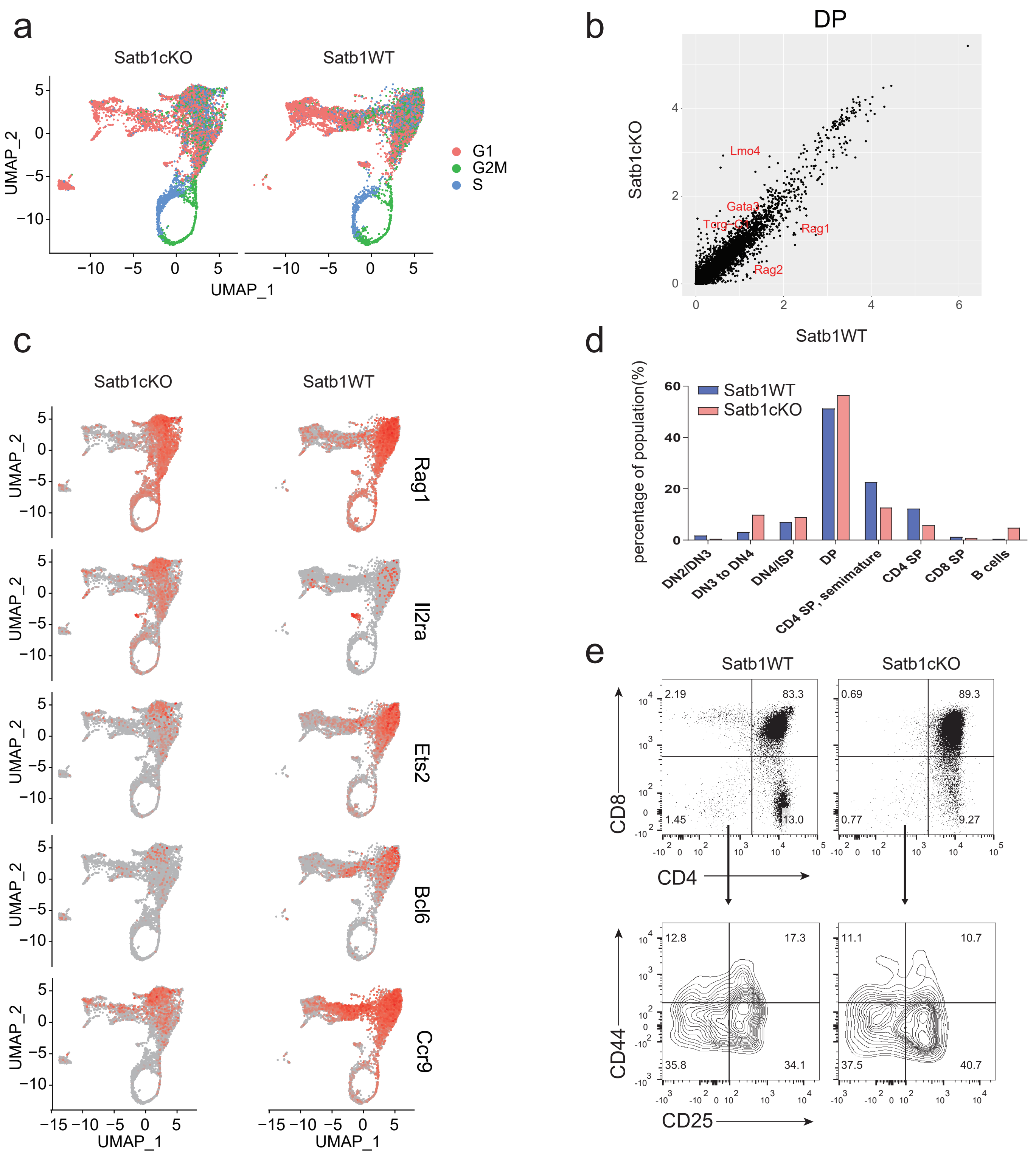
a
Satb1cKO
Satb1WT
UMAP_2
UMAP_1
G1
G2M
S
b
DP
Satb1cKO
Satb1WT
Lmo4
Gata3
Rag1
Rag2
c
Satb1cKO
Satb1WT
Rag1
Il2ra
Ets2
Bcl6
Ccr9
d
percentage of population(%)
Satb1WT
Satb1cKO
DN2/DN3
DN3 to DN4
DN4/ISP
DP
CD4 SP , semimature
CD4 SP
CD8 SP
B cells
e
Satb1WT
Satb1cKO
CD8
CD4
2.19
83.3
1.45
13.0
0.69
89.3
0.77
9.27
CD44
CD25
12.8
17.3
35.8
34.1
11.1
10.7
37.5
40.7

Supplementary Fig. 1 Satb1 controls cell identity of DP thymocytes

a) UMAP plot of thymocytes by cell-cycle phase gene expression. G1, G1 phase; G2M, G2/M phase; S, S phase. Left: Satb1cKO (vav-cre^{+} Satb1$^{fl/fl}$); right: Satb1WT (Satb1$^{fl/fl}$).

b) Dot plot showing the average expression of genes detected in Satb1WT (Satb1$^{fl/fl}$) and Satb1cKO (vav-cre^{+} Satb1$^{fl/fl}$) DP population from the single-cell dataset. There are 383 upregulated and 317 downregulated genes with adjusted p value less than 0.05.

c) UMAP expression plots of representative genes for DP thymocytes (Rag1, Il2ra, Bcl6, Ets2, and Ccr9) between Satb1WT and Satb1cKO group. Left: Satb1cKO (vav-cre^{+} Satb1$^{fl/fl}$); right: Satb1WT (Satb1$^{fl/fl}$).

d) Cell number percentages of thymocyte subsets from the single-cell dataset. Source data are provided as a Source Data file.

e) Flow cytometry of thymocytes from Satb1WT and Satb1cKO mice.

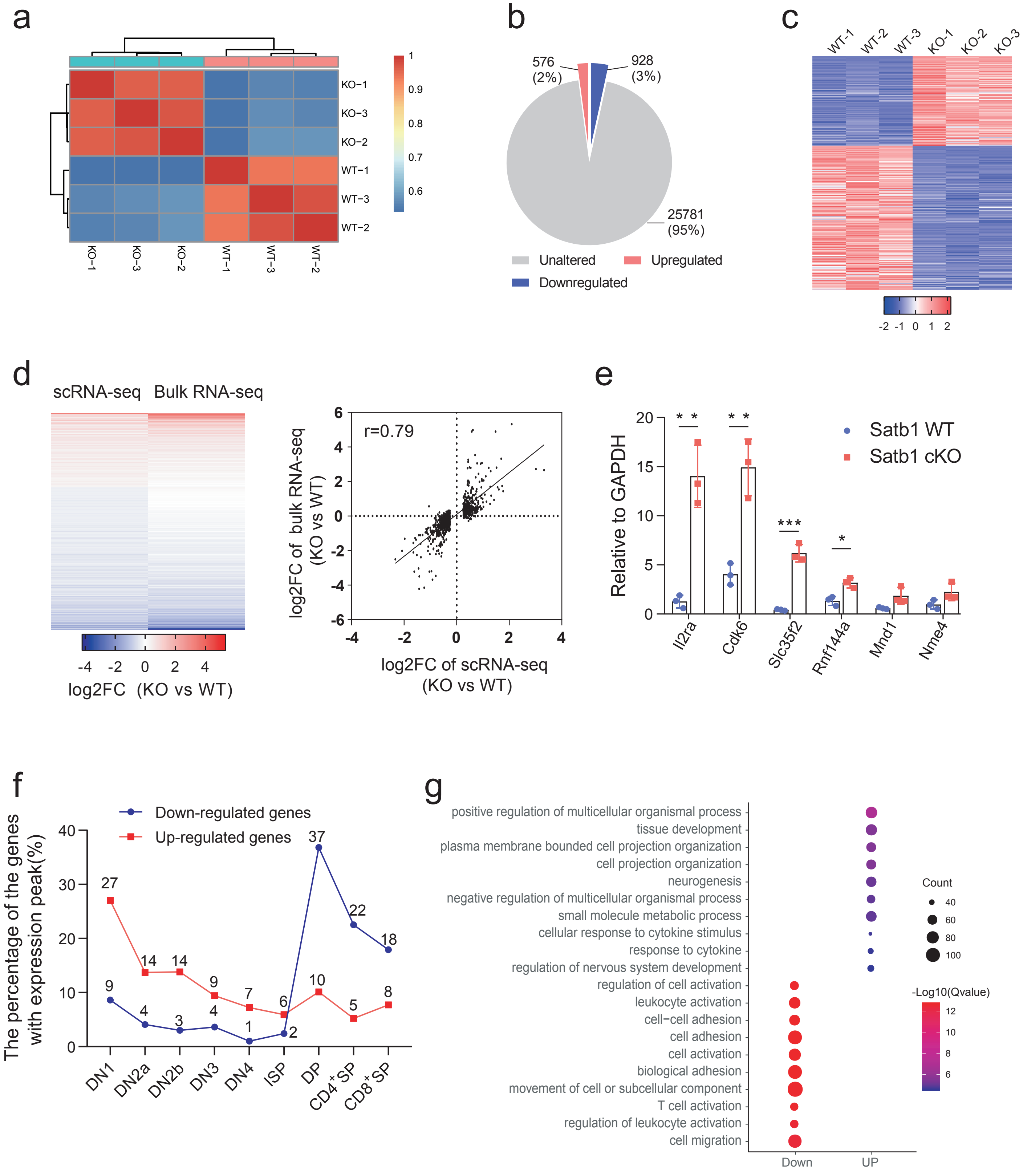

a
KO−1
KO−3
KO−2
WT−1
WT−3
WT−2
1
0.9
0.8
0.7
0.6
b
576
(2%)
928
(3%)
25781
(95%)
Unaltered
Upregulated
Downregulated
c
WT-1
WT-2
WT-3
KO-1
KO-2
KO-3
-2 -1 0 1 2
d
scRNA-seq
Bulk RNA-seq
-4 -2 0 2 4
log2FC (KO vs WT)
r=0.79
log2FC of bulk RNA-seq (KO vs WT)
log2FC of scRNA-seq (KO vs WT)
e
Relative to GAPDH
Satb1 WT
Satb1 cKO
Il2ra
Cdk6
Slc35f2
Rnf144a
Mnd1
Nme4
f
The percentage of the genes with expression peak(%)
Down-regulated genes
Up-regulated genes
DN1
DN2a
DN2b
DN3
DN4
ISP
DP
CD4+SP
CD8+SP
g
positive regulation of multicellular organismal process
tissue development
plasma membrane bounded cell projection organization
cell projection organization
neurogenesis
negative regulation of multicellular organismal process
small molecule metabolic process
cellular response to cytokine stimulus
response to cytokine
regulation of nervous system development
regulation of cell activation
leukocyte activation
cell−cell adhesion
cell adhesion
cell activation
biological adhesion
movement of cell or subcellular component
T cell activation
regulation of leukocyte activation
cell migration
Down
UP
Count
40
60
80
100
-Log10(Qvalue)
12
10
8
6

Supplementary Fig. 2 Satb1 regulates gene expression of DP thymocytes
a) Bulk RNA-seq replicates were hierarchically clustered according to gene expression sample distances using DESeq2. Three biological replicates of each sample were analyzed. WT, Satb1$^{fl/fl}$; KO, *vav*-cre^{+} Satb1$^{fl/fl}$. Source data are provided as a Source Data file.
b) Pie chart indicating the fractions and absolute numbers of differentially expressed genes derived from the analysis of the transcriptome of Satb1 WT and Satb1cKO cells by bulk RNA-Seq. The RNA-Seq analysis indicated that 576 (red) of the total 27,285 genes showed significantly up-regulated (FDR < 0.05) whilst 926 genes were down-regulated (blue). Source data are provided as a Source Data file.
c) Heatmap of differentially expressed genes between Satb1WT and Satb1cKO DP thymocytes. The expression data are normalized log2 fold change. Three biological replicates of bulk RNA-seq data were analyzed. Red represents the up-regulated genes, while blue represents the down-regulated genes. Source data are provided as a Source Data file.
d) Differential expression heatmap (left) and correlation analysis (right) of overlapped differentially expressed genes from scRNA-seq (DP population) and bulk RNA-seq. Source data are provided as a Source Data file.
e) Quantitative PCR to validate the expression changes of down-regulated genes in Satb1cKO DP thymocytes. The data plotted as mean ± SD of three experiments, each with one mouse per genotype. * P < 0.05, ** P < 0.01, **** P < 0.0001 by two-sided Student's *t* test. *Il2ra*, *p*=0.002394; *Cdk6*, *p*=0.003669; *Slc35f2*, *p*=0.000374; *Rnf144a*, *p*=0.010136; *Mnd1*, *p*=0.061639; Nme4, *p*=0.091416. Source data are provided as a Source Data file.
f) Percentage of the differentially expressed genes with an expression peak at each stage during the differentiation of DN1 into SP thymocytes. More than 70% of the down-regulated genes have an expression peak in DP and SP stages, while less one quarter of up-regulated genes in DP and SP stages. Gene expression data from DN1 to SP stages are derived RNA-seq data from ImmGen datasets (GSE109125). Source data are provided as a Source Data file.
g) Gene ontology analysis of the biological processes overrepresented in the group of transcripts up-regulated or down-regulate gene. Top 10 of biological processes were presented here. The size of a circle indicates the number of enriched genes, and its color corresponds to the adjusted p value. *P* values were determined by one-sided hypergeometric tests. FDR corrected *p* values were corrected for multiple testing using the Benjamini and Hochberg method and represented by *q* values. Source data are provided as a Source Data file.

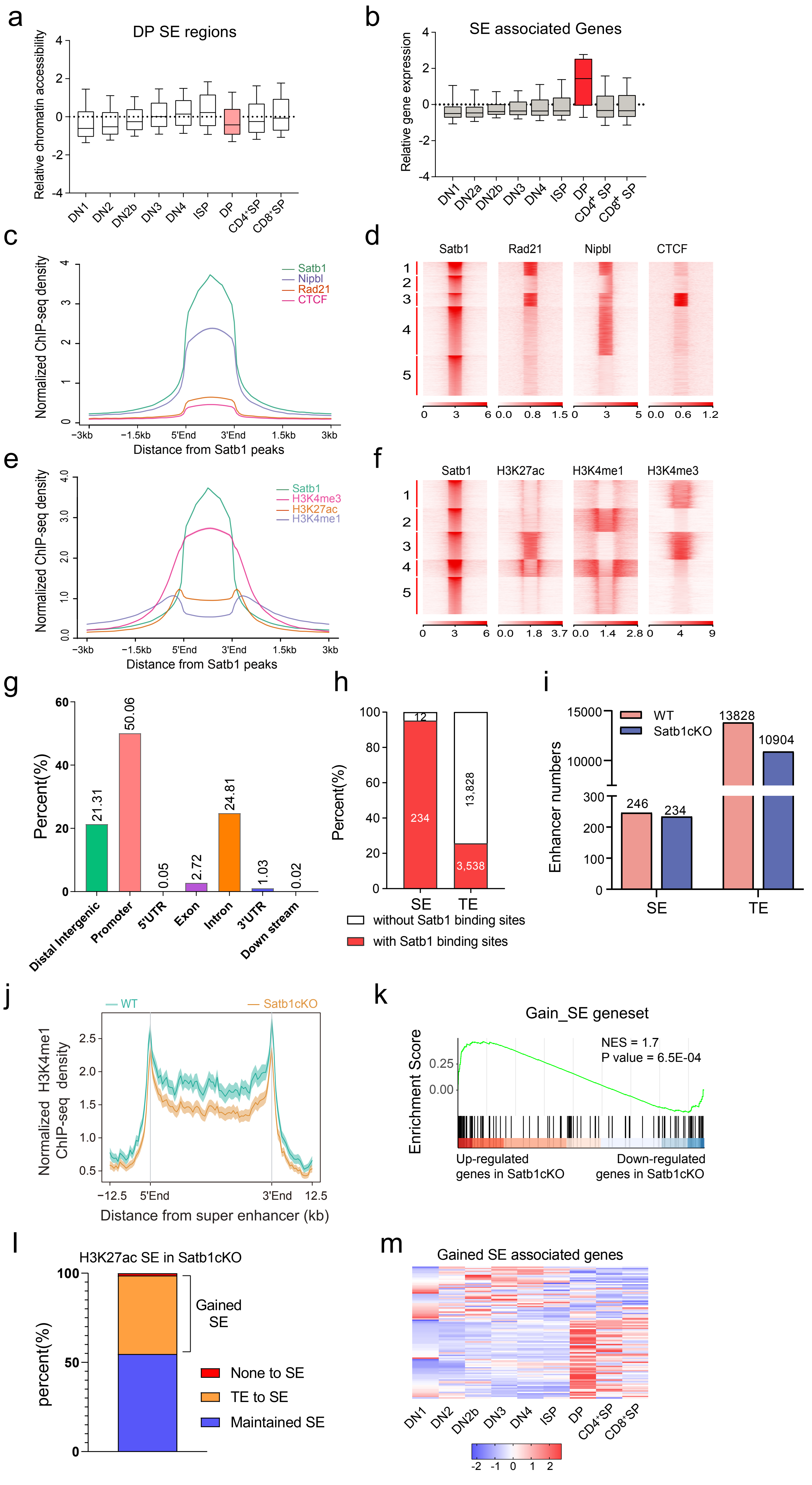

a
DP SE regions
Relative chromatin accessibility
DN1
DN2
DN2b
DN3
DN4
ISP
DP
CD4+SP
CD8+SP
b
SE associated Genes
Relative gene expression
DN1
DN2a
DN2b
DN3
DN4
ISP
DP
CD4+ SP
CD8+ SP
c
Normalized ChIP-seq density
Satb1
Nipbl
Rad21
CTCF
−3kb
−1.5kb
5'End
3'End
1.5kb
3kb
Distance from Satb1 peaks
d
Satb1
Rad21
Nipbl
CTCF
e
Normalized ChIP-seq density
Satb1
H3K4me3
H3K27ac
H3K4me1
Distance from Satb1 peaks
f
Satb1
H3K27ac
H3K4me1
H3K4me3
g
Percent(%)
21.31
50.06
0.05
2.72
24.81
1.03
0.02
Distal Intergenic
Promoter
5'UTR
Exon
Intron
3'UTR
Down stream
h
Percent(%)
12
234
13,828
3,538
SE
TE
without Satb1 binding sites
with Satb1 binding sites
i
Enhancer numbers
WT
Satb1cKO
13828
10904
246
234
SE
TE
j
WT
Satb1cKO
Normalized H3K4me1 ChIP-seq density
−12.5
5'End
3'End
12.5
Distance from super enhancer (kb)
k
Gain_SE geneset
Enrichment Score
NES = 1.7
P value = 6.5E-04
Up-regulated genes in Satb1cKO
Down-regulated genes in Satb1cKO
l
H3K27ac SE in Satb1cKO
percent(%)
Gained SE
None to SE
TE to SE
Maintained SE
m
Gained SE associated genes
DN1
DN2
DN2b
DN3
DN4
ISP
DP
CD4+SP
CD8+SP

Supplementary Fig. 3 Related to Figure 2

a) Box plot of chromatin accessibility in the SE regions (3704 ATAC-seq peaks) and **b**) expression of SE-associated genes during thymocyte development from DN1 to SPs. Box plots show median (center line), interquartile range (box) and tenth and ninetieth percentiles (whiskers). Chromatin accessible signals and gene expression are from ATAC-seq data and RNA-seq data of ImmGen datasets. Source data are provided as a Source Data file.

c) Line diagram and **d**) heatmap of Rad21, Nipbl, and CTCF ChIP-seq signal intensity at Satb1 binding sites (top 10000 regions) in DP thymocytes. ChIP-seq data are same as Fig. 2c.

e) Line diagram and **f**) heatmap of H3K27ac, H3K4me1, and H3K4me3 ChIP-seq signal intensity at Satb1 binding sites. ChIP-seq data are same as Fig. 2c.

g) Distribution of Satb1 binding sites (top 10000 regions) in mouse DP thymocytes. Source data are provided as a Source Data file.

h) Bar graph showing the percentage of super-enhancers (SE) or traditional enhancers (TE) occupied by Satb1 or not. Source data are provided as a Source Data file.

i) Bar graph showing the SE or TE numbers of WT and Satb1cKO DP thymocytes. The Satb1cKO SEs were called from the H3K27 acetylation ChIP-seq data of Satb1cKO DP thymocytes using the same method as the WT SEs. ChIP-seq data are same as Fig. 2c. Source data are provided as a Source Data file.

j) Line plot displaying H3K4me1 intensity in Satb1WT vs Satb1cKO at WT DP super-enhancer regions. Data represent the mean ± SEM.

k) GSEA enrichment plot of the differentially expressed genes enriched for the gained super-enhancer associated geneset.The DEGs are from bulk RNA-seq data. Nominal p value, calculated as two-sided t-test, no adjustment since only one gene set was tested. Source data are provided as a Source Data file.

l) Percentage distribution of the SEs in Satb1 deficient DP thymocytes. Maintained SEs, SEs in wild type DP thymocytes; TE to SE, SATB1cKO-SE regions are TEs in wild type DP thymocytes; None to SE, the regions are neither SEs nor TEs in wild type DP thymocytes. Source data are provided as a Source Data file.

m) Heatmap showing relative expression of gained-SE associated genes from DN1 to SP stages. Expression levels are represented as Z-scores. Gene expression data are derived from RNA-seq data of ImmGen genesets. Source data are provided as a Source Data file.

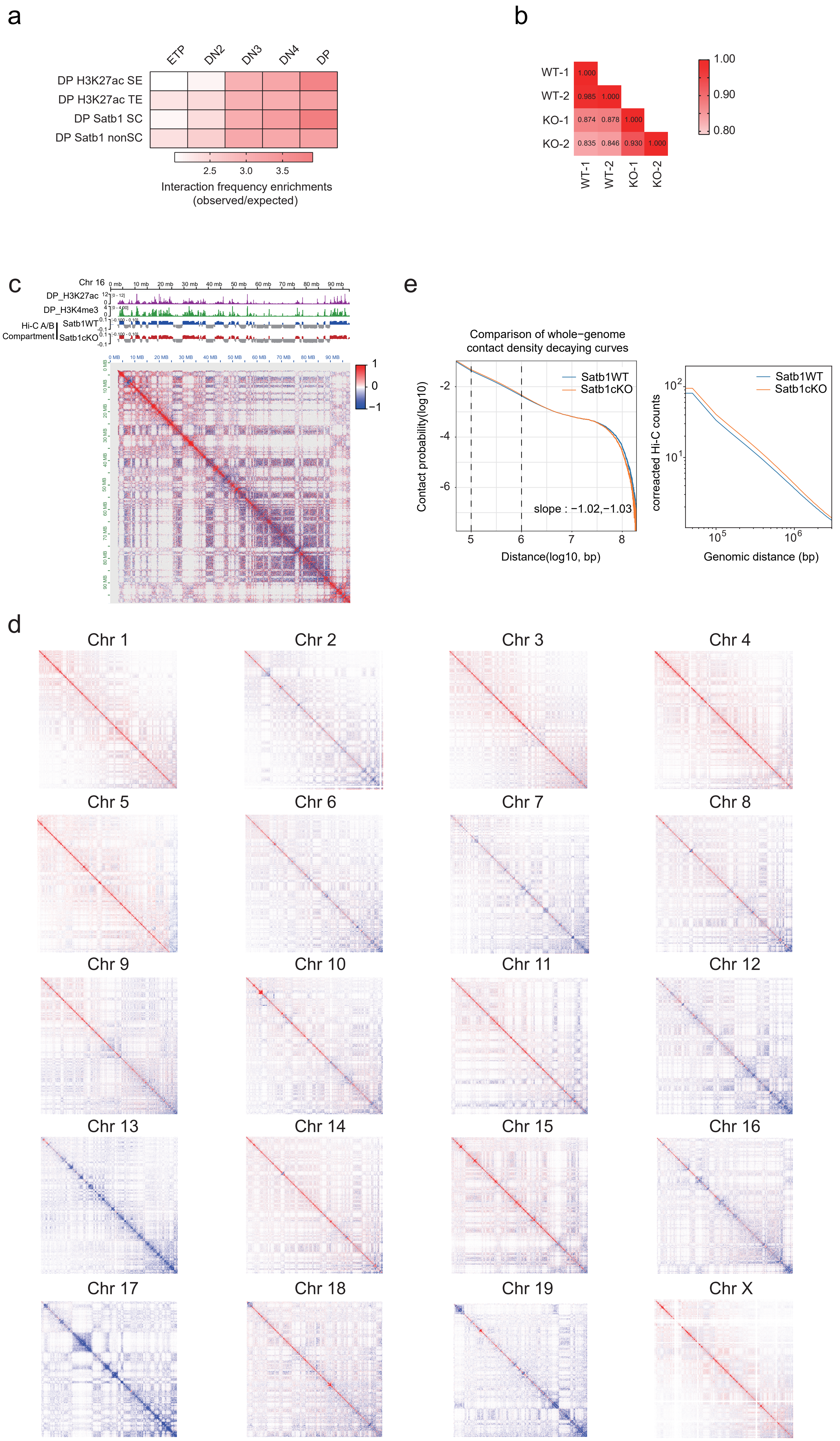
a
ETP
DN2
DN3
DN4
DP
DP H3K27ac SE
DP H3K27ac TE
DP Satb1 SC
DP Satb1 nonSC
2.5
3.0
3.5
Interaction frequency enrichments
(observed/expected)
b
WT-1
WT-2
KO-1
KO-2
1.000
0.985
0.874
0.878
0.835
0.846
0.930
1.00
0.90
0.80
c
Chr 16
DP_H3K27ac
DP_H3K4me3
Hi-C A/B Compartment
Satb1WT
Satb1cKO
1
0
−1
d
Chr 1
Chr 2
Chr 3
Chr 4
Chr 5
Chr 6
Chr 7
Chr 8
Chr 9
Chr 10
Chr 11
Chr 12
Chr 13
Chr 14
Chr 15
Chr 16
Chr 17
Chr 18
Chr 19
Chr X
e
Comparison of whole−genome
contact density decaying curves
Satb1WT
Satb1cKO
slope : −1.02,−1.03
Contact probability(log10)
Distance(log10, bp)
correacted Hi-C counts
Genomic distance (bp)

Supplementary Fig. 4 Related to Figure 4

a) Heatmap showing changes in chromatin contacts in H3K27ac-SE, H3K27ac-TE, Satb1-SC, and Satb1-nonSC regions from ETP to DP stages. Chromatin interaction signals are from the published 3e Hi-C data (GSE79422)[36]. Interaction frequency enrichment was observed paired-end tags (PETs) divided by expected PETs. Source data are provided as a Source Data file.

b) Pair-wise Pearson correlation coefficient between wild type and Satb1cKO replicates (top 1000 variables bins). The scores of Pearson correlation coefficient are showed in different degrees of red. Source data are provided as a Source Data file.

c) Subtraction heatmap of Hi-C interaction matrix from Juicebox corresponding to Satb1cKO–Satb1WT at 500 kb resolution for chromosome 16, with H3K27ac and H3K4me3 ChIP-seq and A/B compartment signals on top.

d) Subtraction heatmap (corresponding to Satb1cKO–Satb1WT) of 10kb binned Hi-C data of DP thymocytes from WT or Satb1cKO mice. Data are one of two independent experiments.

e) Chromatin contact frequency decay curves of control (blue) and Satb1cKO (red) Hi-C data.

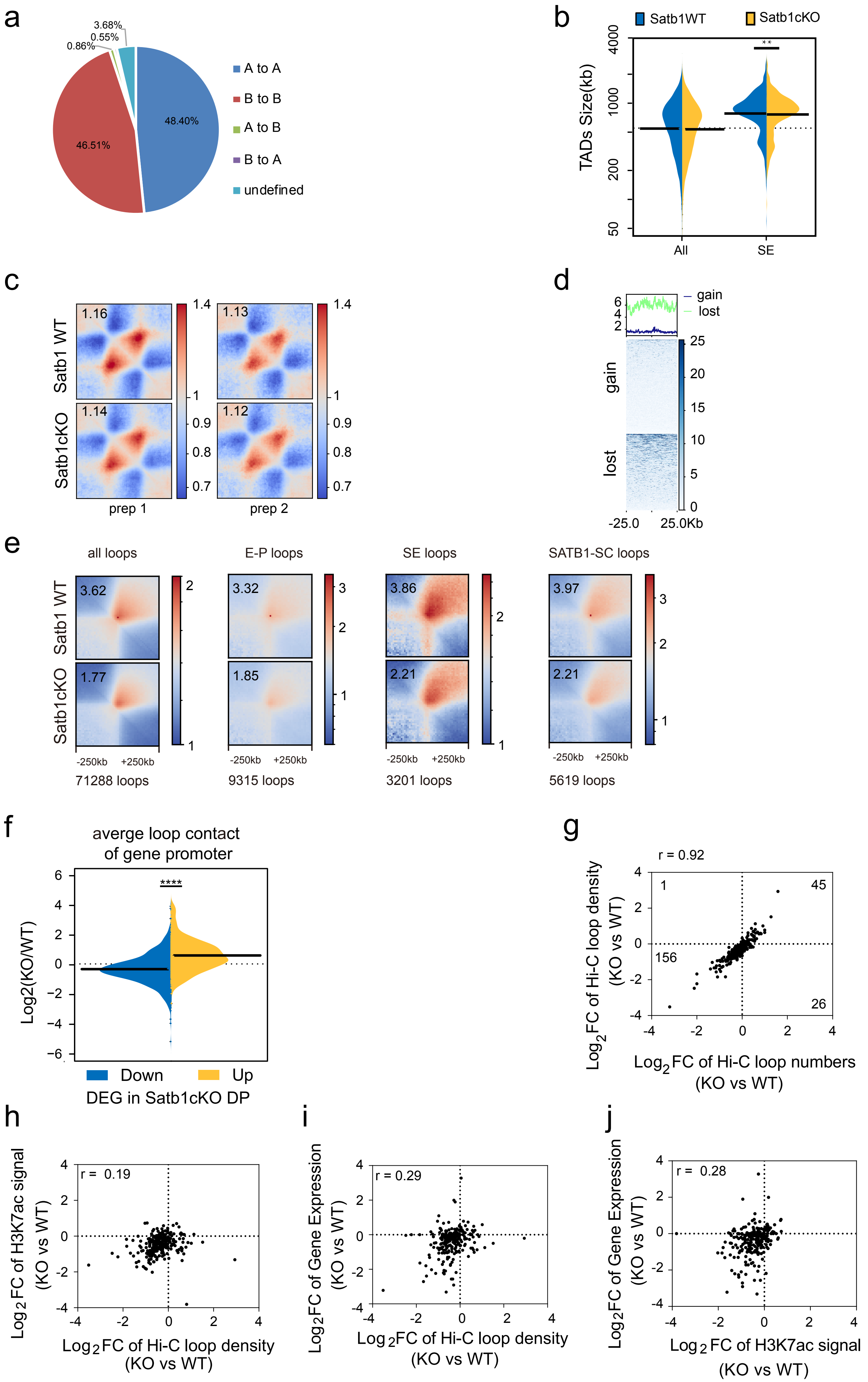
a
3.68%
0.55%
0.86%
48.40%
46.51%
A to A
B to B
A to B
B to A
undefined
b
Satb1WT
Satb1cKO
TADs Size(kb)
4000
1000
200
50
**
All
SE
c
Satb1 WT
Satb1cKO
1.16
1.13
1.14
1.12
1.4
1
0.9
0.8
0.7
prep 1
prep 2
d
gain
lost
6
4
2
25
20
15
10
5
0
-25.0
25.0Kb
e
all loops
E-P loops
SE loops
SATB1-SC loops
3.62
1.77
3.32
1.85
3.86
2.21
3.97
2.21
-250kb
+250kb
71288 loops
9315 loops
3201 loops
5619 loops
f
averge loop contact
of gene promoter
Log2(KO/WT)
****
Down
Up
DEG in Satb1cKO DP
g
r = 0.92
1
45
156
26
Log2FC of Hi-C loop density
(KO vs WT)
Log2FC of Hi-C loop numbers
(KO vs WT)
h
r = 0.19
Log2FC of H3K7ac signal
(KO vs WT)
Log2FC of Hi-C loop density
(KO vs WT)
i
r = 0.29
Log2FC of Gene Expression
(KO vs WT)
Log2FC of Hi-C loop density
(KO vs WT)
j
r = 0.28
Log2FC of Gene Expression
(KO vs WT)
Log2FC of H3K7ac signal
(KO vs WT)

Supplementary Fig. 5 Related to Figure 4

a) Pie chart showing the compartment changes between Satb1WT and Satb1cKO DP. A to A and B to B, compartment regions kept in Satb1cKO DP thymocytes. A to B, compartment A regions changed to compartment B in Satb1cKO; B to A, compartment B regions changed to compartment A in Satb1cKO. Undefined, unable to define compartment states in either wild type or Satb1cKO. Source data are provided as a Source Data file.

b) Split violin plots correspond to sizes of total or super-enhancer (SE) containing TADs from Hi-C data of WT or Satb1cKO thymocytes. * $p < 0.05$, ** $p < 0.01$ by Mann Whitney U test for all TADs or two-side Student's t test for SE TADs. Two sided Mann Whitney U test for all TADs (p=0.0498) and paired *t* test for SE groups (p=0.0036). Source data are provided as a Source Data file.

c) Aggregate TAD analysis for WT (up) and Satb1cKO (bottom) DP thymocytes. Average coverage-corrected Hi-C contact matrices are shown centered around 2 × 495kb long TADs identified in the WT thymocytes.

d) Line plots and heatmap of H3K27ac signals in gained and lost SE regions in wild type DP thymocytes. H3K27ac signals are from ChIP-seq data of DP thymocytes published previously (DRP003376)[28].

e) A biological replicate related to Figure 4c. Pile-up plots showing contacts of ± 250kb regions around the loops in WT or Satb1cKO Hi-C (10 kb resolution).

f) Split violin plots for average loop contacts in promoters of the genes down-regulated (blue) or up-regulated (yellow) in Satb1cKO thymocytes. Down-regulated gene, n=601; Up-regulated gene, n=368; *p*=2.20e-37. **** $p < 0.0001$ by two-sided Student's t test. Source data are provided as a Source Data file.

g-j) Pearson correlation analysis of super-enhancer associated **g**) loop numbers with average loop contacts, **h**) average loop contacts with H3K27ac signals, **i**) average loop contacts with gene expression, and **j**) H3K27ac signals with gene expression. Source data are provided as a Source Data file.

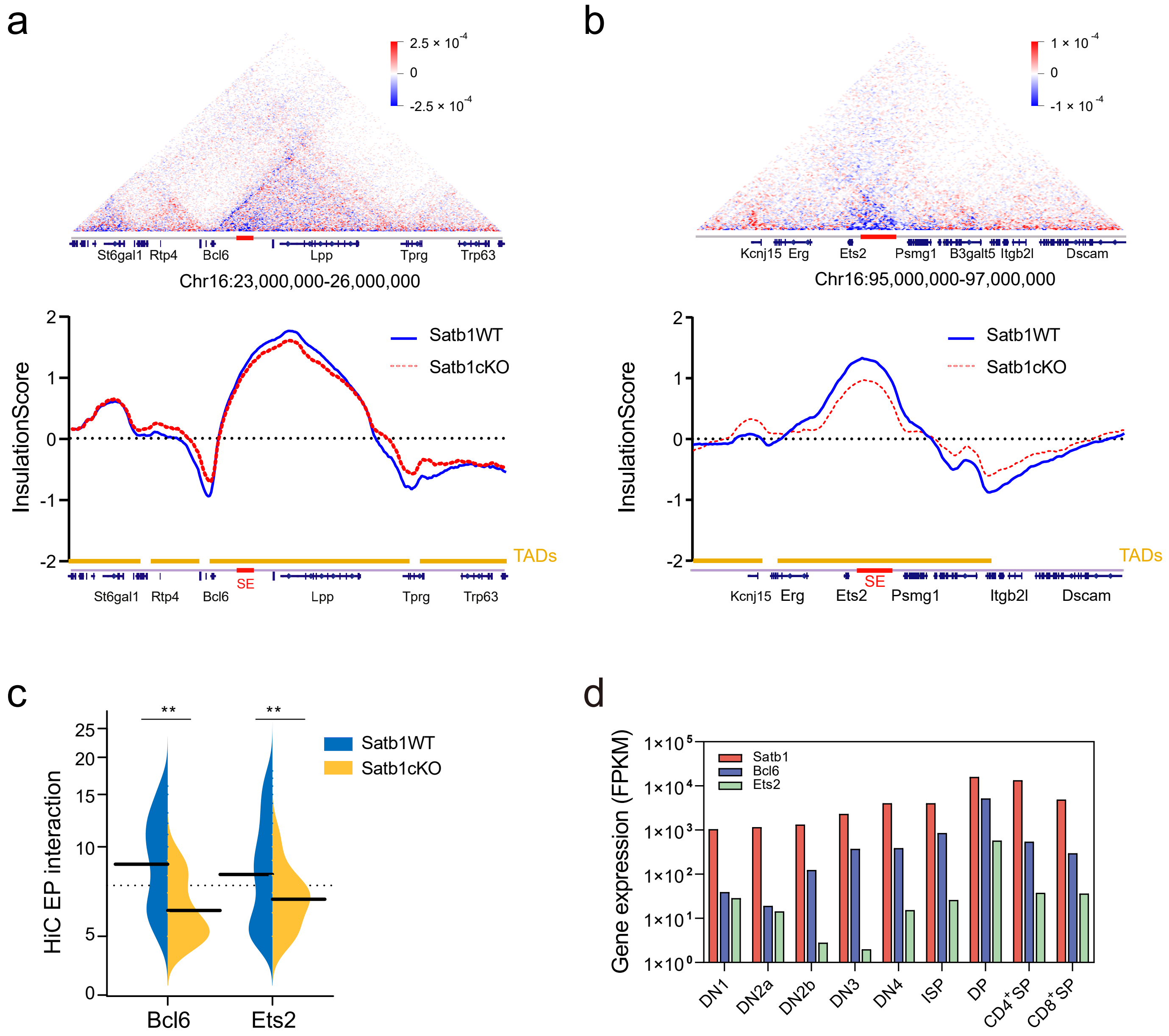
a
2.5 × 10-4
0
-2.5 × 10-4
St6gal1 Rtp4 Bcl6 Lpp Tprg Trp63
Chr16:23,000,000-26,000,000
InsulationScore
Satb1WT
Satb1cKO
TADs
SE
b
1 × 10-4
0
-1 × 10-4
Kcnj15 Erg Ets2 Psmg1 B3galt5 Itgb2l Dscam
Chr16:95,000,000-97,000,000
InsulationScore
Satb1WT
Satb1cKO
TADs
SE
c
HiC EP interaction
Satb1WT
Satb1cKO
Bcl6
Ets2
d
Gene expression (FPKM)
Satb1
Bcl6
Ets2
DN1 DN2a DN2b DN3 DN4 ISP DP CD4+SP CD8+SP

Supplementary Fig. 6 Related to figure 5
a) and **b**) Subtraction heatmaps of Hi-C interactions (up) and insulation score profiles (bottom) of Hi-C data at **a**) Bcl6 and **b**) Ets2 loci in WT (red) and Satb1cKO (blue) DP thymocytes. TADs are yellow lines. Source data are provided as a Source Data file.

c) Split violin plots for enhancer-promoter (EP) interactions of the Bcl6 and Ets2 loci in WT and Satb1cKO DP thymocytes. *Bcl6*, p=0.0023; *Ets2*, p=0.0019. **p value < 0.01 by Mann-Whitney test. Source data are provided as a Source Data file.

d) The expression values (FPKM) of Satb1, Bcl6, and Ets2 during the differentiation of DN1 into SP thymocytes. Expression data are derived from RNA-seq of ImmGen datasets. Source data are provided as a Source Data file.

a

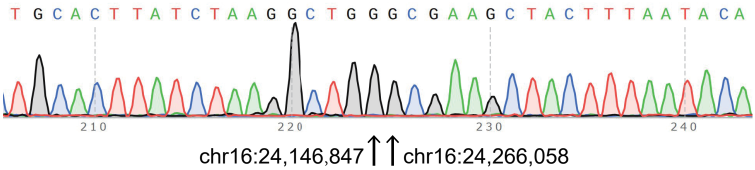
T G C A C T T A T C T A A G G C T G G G C G A A G C T A C T T T A A T A C A
210
220
230
240
chr16:24,146,847
chr16:24,266,058

b

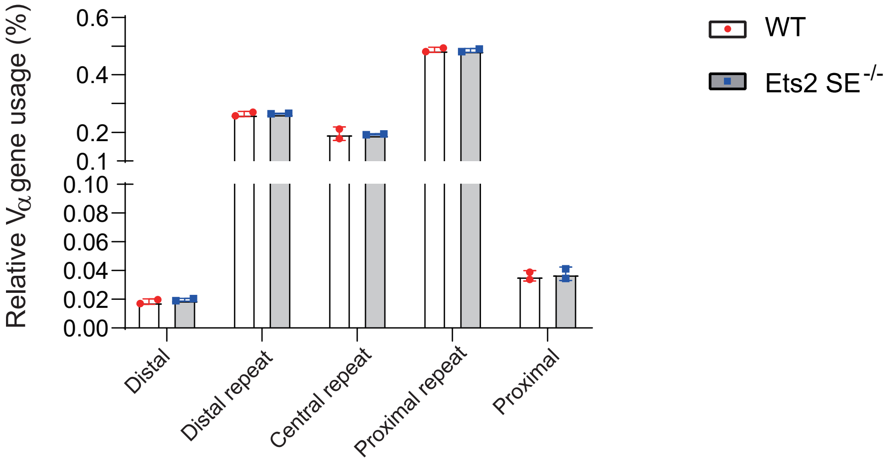
Relative Vα gene usage (%)
0.6
0.4
0.2
0.1
0.10
0.08
0.06
0.04
0.02
0.00
Distal
Distal repeat
Central repeat
Proximal repeat
Proximal
WT
Ets2 SE-/-

c

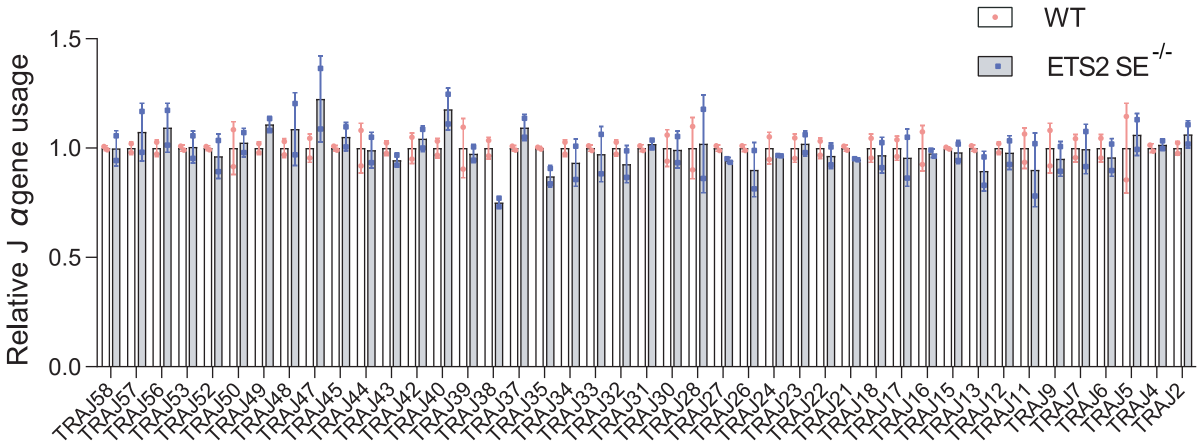
Relative J α gene usage
1.5
1.0
0.5
0.0
WT
ETS2 SE-/-
TRAJ58
TRAJ57
TRAJ56
TRAJ53
TRAJ52
TRAJ50
TRAJ49
TRAJ48
TRAJ47
TRAJ45
TRAJ44
TRAJ43
TRAJ42
TRAJ40
TRAJ39
TRAJ38
TRAJ37
TRAJ35
TRAJ34
TRAJ33
TRAJ32
TRAJ31
TRAJ30
TRAJ28
TRAJ27
TRAJ26
TRAJ24
TRAJ23
TRAJ22
TRAJ21
TRAJ18
TRAJ17
TRAJ16
TRAJ15
TRAJ13
TRAJ12
TRAJ11
TRAJ9
TRAJ7
TRAJ6
TRAJ5
TRAJ4
TRAJ2

d

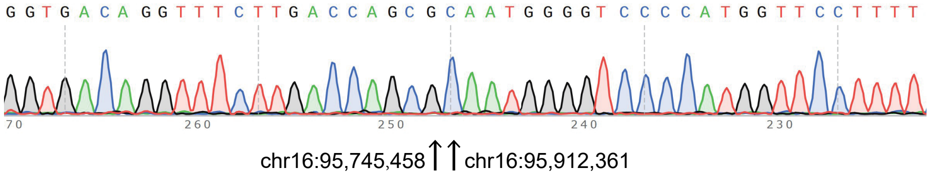
G G T G A C A G G T T T C T T G A C C A G C G C A A T G G G G T C C C C A T G G T T C C T T T T
70
260
250
240
230
chr16:95,745,458
chr16:95,912,361

Supplementary Fig. 7 Ets2 SE deletion doesn't affect Tcra rearrangement

a) Sanger sequencing chromatogram showing the joint sequence of the Ets2-SE deletion. CRISPR-Cas9 system was used to delete the Est2-SE region from chr16:24,146,848 to chr16:24,266,057. The primers were designed at the upstream and downstream of the rejoined site and PCR products were Sanger-sequenced.

b) and **c**) Relative Vα and Jα usages determined by deep-sequencing of *Tcra* transcripts amplified by 5'RACE of WT and Ets2 $SE^{-/-}$ thymocytes, respectively. The relative Vα or Jα usages were calculated by dividing the number of the clonotypes containing the Vα or Jα genes by the total clonotype number. n=2 biologically independent experiments. Data represent the mean ± SD. Source data are provided as a Source Data file.

d) Sanger sequencing chromatogram showing the joint sequence of the Bcl6-SE deletion. CRISPR-Cas9 system was used to delete the Bcl6-SE region from chr16:95,745,459 to chr16:95,912,360.

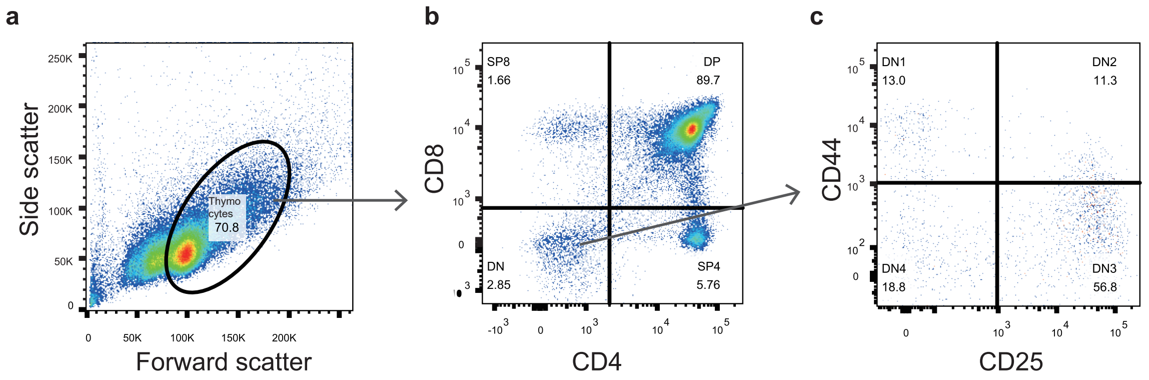
a
Side scatter
250K
200K
150K
100K
50K
0
Thymo cytes
70.8
0
50K
100K
150K
200K
Forward scatter
b
SP8
1.66
DP
89.7
DN
2.85
SP4
5.76
CD8
CD4
c
DN1
13.0
DN2
11.3
DN4
18.8
DN3
56.8
CD44
CD25

Supplemental fig. 8 Gating of DP and DN cells. a) Cells were gated in FSC vs SSC dot plot. b) The thymocytes were further gated based on CD4 and CD8 expression as SP4 ($CD4^{+}CD8^{-}$), DP ($CD4^{+}CD8^{+}$), SP8 ($CD4^{-}CD8^{+}$) and DN ($CD4^{-}CD8^{-}$). c) CD25 and CD44 were used as markers for DN subpopulations.
