# Supplementary material for: Chromatin organizer SATB1 controls the cell identity of CD4+ CD8+ double-positive thymocytes by regulating the activity of super-enhancers
Source: Nat Commun. 2022 Sep 22;13:5554. doi: 10.1038/s41467-022-33333-6 (PMC9500044; doi:10.1038/s41467-022-33333-6)
Supplement: Supplementary file 3 — Description of Additional Supplementary Files [file 41467_2022_33333_MOESM3_ESM.pdf]

Description of Additional Supplementary Files

Supplementary Data S1

Description: sheet1, scRNA-seq differentially expressed genes of cluser 0 and cluster 3; sheet2, scRNA-seq differentially expressed genes of DP population from wild type and Satb1 deleted mice. (pct.1: The percentage of cells where the feature is detected in the Satb1 KO group; pct.2: The percentage of cells where the feature is detected in wild type group.); sheet3, Bulk RNA-seq differentially expressed genes of sorted DP cells from wild type and Satb1 deleted mice.

Supplementary Data S2

Description: The DN1 and DN3 genesets used in Fig. 1f.

Supplementary Data S3

Description: sheet1, Gene ontology enrichment of down-regulated genes of bulk RNA-seq data; sheet2, Gene ontology enrichment of up-regulated genes of bulk RNA-seq data.

Supplementary Data S4

Description: sheet1, super-enhancers and the associated genes in wild type DP thymocytes; sheet2, super-enhancers and the associated genes in Satb1-cKO DP thymocytes.

Supplementary Data S5

Description: SATB1 super-clusters and the associated genes.

Supplementary Data S6

Description: sheet1, data used in Fig. 1d; sheet2, data used in Fig. 2b; sheet3, data used in Fig. 2h; sheet4, data used in Fig. 3e; sheet5, data used in Fig. S2c.
