# Supplementary material for: Chromatin organizer SATB1 controls the cell identity of CD4+ CD8+ double-positive thymocytes by regulating the activity of super-enhancers
Source: Nat Commun. 2022 Sep 22;13:5554. doi: 10.1038/s41467-022-33333-6 (PMC9500044; doi:10.1038/s41467-022-33333-6)
Supplement: Supplementary file 11 — Source Data [file 41467_2022_33333_MOESM11_ESM.zip › data source information.docx]

Figure 1c. Differentially expressed genes of DP cells from WT and SATB1 cKO mice by using bulk RNA-seq.

Figure 1d and 1e. Relative gene expression of the differentially expressed genes during the differentiation from DN1 to SP thymocytes.

Figure 1f. DN1 and DN3 genesets were generated according to the highest expression stage from DN1 to SP stages.

Figure 2a. SEs, TEs, and their associated genes based on normalized H3K27ac signals and rank in Satb1WT DP thymocytes using the ROSE algorithm.

Figure 2b. Relative expression of SE-associated genes during the differentiation from DN1 to SP thymocytes.

Figure 2f. Gained, maintained, and lost SEs.

Figure 2g. The geneset of the differentially expressed genes enriched for the WT DP super-enhancer associated geneset and the Satb1cKO DP lost super-enhancer associated geneset.

Figure 2h. The expression of WT DP super-enhancer-associated genes in WT and Satb1cKO DP thymocytes.

Figure 2i. Relative expression of genes associated with super-enhancers gained, maintained, or lost in Satb1 KO DP.

Figure 2j. Relative expression of the super-enhancer (SE) or traditional enhancer (TE) associated genes in Satb1cKO DP cells.

Figure 3a. Clusters ranked by normalized Satb1 ChIP-seq signals in DP thymocytes using the ROSE algorithm.

Figure 3b. Satb1 super-clusters and H3K27ac super-enhancers in WT DP thymocytes.

Figure 3d. Satb1 super-cluster associated genes.

Figure 3e and Figure S3b. Relative gene expressions of SATB1 super-culster-associated genes during the differentiation from DN1 to SP thymocytes.

Figure 3f. Gene ontology analysis on the genes associated with super-enhancers by enrichment for H3K27ac or super-clusters of Satb1.

Figure 4a. The contact distances between two anchors of significantly changed contacts (50kb bin) in Satb1 deficient DP thymocytes.

Figure 4b. Satb1, H3K27ac, and CTCF ChIP-seq signals in anchors of differential chromatin interactions (50kb bin).

Figure 4d. Loop sizes of all loops, enhancer-promoter (E-P) loops, super-enhancer (SE) loops, and Satb1-SC loops identified from Hi-C data of Satb1WT thymocytes.

Figure 4e. Pearson’s correlation analysis of super-enhancer associated H3K27ac signals (left) or relative gene expression (right) with numbers of loops associated with WT super-enhancers.

Figure 5e. Relative expression of Bcl6 and Est2 in Satb1WT and Satb1cKO DP thymocytes detected by reverse-transcripted quantitative PCR.

Figure 5f. Western blot showing protein expression of Satb1, Bcl6, and Ets2 in WT and Satb1cKO thymocytes.

Figre 5g. The expression profiles of Satb1, Bcl6, and Ets2 during the differentiation of DN1 into DP thymocytes.

Figure 6a. Relative Ets2 expression in thymocytes from Ets2 super-enhancer knockout (Ets2 SE-/-) and wild-type (WT) mice was analyzed using reverse-transcription qPCR.

Figure 6d. Cell numbers of wild-type and Ets2-SE knockout thymi.

Figure 6e. The cell viability assay.

Figure 6f. Flow cytometry of thymocytes from WT and Ets2 SE-/- mice.

Figure 7a. Relative Bcl6 expression in thymocytes.

Figure 7c. Western blot showing Bcl6 protein expression.

Figure 7d. Cell numbers of thymi from WT, Bcl6 SE heterozygous, and Bcl6 SE homozygous mice.

Figure 7e. Flow cytometry of thymocytes from WT and Bcl6 SE-/- mice.

Figure 7f. The cell viability assay.

Figure 7f Relative Vα usages were determined by deep-sequencing of Tcra transcripts amplified by 5’RACE of WT and Bcl6 SE-/- thymocyte.

Figure 7h. Relative Jα usages were determined by deep-sequencing of Tcra transcripts amplified by 5’RACE of WT and Bcl6 SE-/- thymocyte.

Figure S1d. Cell number percentages of thymocyte subsets from the single-cell dataset.

Figure S2a. Bulk RNA-seq replicates.

Figure S2b. Differentially expressed genes derived from the analysis of the transcriptome of Satb1 WT and Satb1cKO cells by bulk RNA-Seq.

Figure S2c. Differentially expressed genes between Satb1WT and Satb1cKO DP thymocytes.

Figure S2d. Differentially expressed genes from scRNA-seq (DP population) and bulk RNA-seq.

Figure s2e. Quantitative PCR to validate the expression changes of down-regulated genes in Satb1cKO DP thymocytes.

Figure S2f. Percentage of the differentially expressed genes with an expression peak at each stage during the differentiation of DN1 into SP thymocytes.

Figure S2g. Gene ontology analysis of the biological processes overrepresented in the group of transcripts up-regulated or down-regulate gene.

Figure S3a. Chromatin accessibility in the SE regions.

Figure S3b. Expression of SE-associated genes.

Figure S3g. Satb1 binding sites (top 10000 regions).

Figure 3h. Super-enhancers (SE) or traditional enhancers (TE).

Figure S3i. the SE or TE numbers of Satb1cKO DP thymocytes.

Figure S3k. The gained super-enhancer associated geneset.

Figure S3l. Percentage distribution of the SEs in Satb1 deficient DP thymocytes.

Figure S3m. Relative expression of gained-SE associated genes from DN1 to SP stages.

Figure S4a. Chromatin contacts in H3K27ac-SE, H3K27ac-TE, Satb1-SC, and Satb1-nonSC regions from ETP to DP stages.

Figure S4b. Compartments.

Figure S5a. Compartment changes.

Figure S5b. Sizes of TADs.

Figure S5f. Average loop contacts in promoters of the genes down-regulated (blue) or up-regulated (yellow) in Satb1cKO thymocytes.

Figure S5g Contacts of SEs.

Figure S5h. H3K27ac signals in SEs

Figure S5i. Expression of SE-associated genes.

Figre S5j. H3K27ac of SEs and expression of SEs-associated gens.

Figure S6a. Insulation scores in the Bcl6 locus.

Figure S6b. Insulation scores in the Ets2 locus.

Figure S6c. Enhancer-promoter (EP) interactions of the Bcl6 and Ets2 loci in WT and Satb1cKO DP thymocytes.

Figure S6d. The expression values (FPKM) of Satb1, Bcl6, and Ets2 during the differentiation of DN1 into SP thymocytes.

Figure S7b. Relative Vα usages determined by deep-sequencing of Tcra transcripts amplified by 5’RACE.

Figure S7c. Relative Jα usages determined by deep-sequencing of Tcra transcripts amplified by 5’RACE.
